# A new minimally invasive technique for correction of pectus carinatum

**DOI:** 10.1186/s13019-021-01663-z

**Published:** 2021-09-28

**Authors:** Wei Ping, Shengling Fu, Yangkai Li, Jun Yu, Ni Zhang, Xiangning Fu, Yixin Cai

**Affiliations:** Department of Thoracic Surgery, Tongji Hospital, Tongji Medical College, Huazhong University of Science and Technology, No. 1095 Jiefang Road, Wuhan, 430030 Hubei Province China

**Keywords:** Pectus carinatum, Minimally invasive correction, Pectus bar, Fixation

## Abstract

**Background:**

The Abramson technique for the correction of pectus carinatum (PC) is commonly performed worldwide. However, the postoperative complications of this technique related to bar fixation, including wire breakage and bar displacement, are relatively high. In this study, a new minimally invasive technique for correction of PC is described, in which the pectus bar is secured by bilateral selected ribs, and for which no special fixation to the rib is needed.

**Methods:**

The procedure was performed by placing the pectus bar subcutaneously over the sternum with both ends of the bar passing through the intercostal space of the selected rib at the anterior axillary line. The protruding sternum was depressed by the bar positioned in this 2 intra- and 2 extra-thorax manners. Between October 2011 and September 2019, 42 patients with PC underwent this procedure.

**Results:**

Satisfactory cosmetic results were obtained in all the patients. The mean operation time was 87.14 min, and the mean postoperative stay was 4.05 days. Wound infection occurred in 3 patients, 2 were cured by antibiotics, and 1 received bar removal 4 months after the initial operation due to the exposure of the implant resulting from uncontrolled infection. Mild pneumothorax was found in 3 patients and cured by conservative treatment. One patient suffered from hydropneumothorax, which was treated with chest drainage. The bars were removed at a mean duration of 24.4 months since primary repair in 20 patients without recurrence.

**Conclusions:**

This new technique for minimally invasive correction of PC deformity is a safe and feasible procedure yielding good results and minimal complications.

## Introduction

Pectus carinatum (PC) is a chest deformity characterized by the excessive protrusion of the sternum and the adjacent costal cartilages. It is one of the most common chest wall deformities, with a reported prevalence of about 0.06%, second only to pectus excavatum [1, 2]. At present, the minimal access procedure for the repair of PC [3], first introduced in 2005 by Abramson, in which a steel bar is placed subcutaneously over the sternum and fixed to the ribs with wires, has been widely applied with good cosmetic results [4–6]. In the Abramson technique, the bar is placed entirely outside the chest wall, and thus all the force on the sternum is sustained by wires. Wire breakage is a common postoperative complication in this procedure, with a reported incidence of 5.2–16.6% [4, 7, 8], and reaching as high as 100% in Lee et al.’s report [9]. It should be noted that early wire breakage is the primary cause of bar displacement that results in orthopedic failure and re-operation [10].

In this article, we describe a new minimally invasive technique for correction of pectus carinatum, in which a modeled pectus-bar is placed over the sternum through the subcutaneous tunnel with both ends passed through the intercostal space of the selected rib at the anterior axillary line. In this way, the bar is driven into and then brought out of the thorax on both sides (2 intra- and 2 extra-thorax) without special fixation to the ribs. The protruding sternum is depressed by force provided by the bilaterally selected ribs exerting force onto the bar without manual pressure.

## Patients and methods

### Patients

From October 2011 to September 2019, 42 patients (39 males and 3 females) with PC underwent the surgical correction in this new procedure at our department. Records of these patients were retrospectively reviewed. Written consent was obtained from patients or from their parents if their age was under 18 years. The study was conducted in accordance with the principles of the Declaration of Helsinki.

The mean age of the patients was 13.69 years (range 5–17 years). All patients presented with chondrogladiolar PC, with 34 being symmetric and 8 being asymmetric. Preoperative examinations were performed on all patients and included laboratory tests, cardiovascular evaluation [electrocardiography (ECG)] and radiological investigations [chest X-ray or computed tomography (CT)]. Evaluation of the chest wall flexibility was routinely performed on all patients by compression of the protruding area with the examiner’s hand with the patient in a supine position or leaning against a wall. There were no abnormal findings on routine preoperative cardiac and pulmonary function evaluations. The mean value of the preoperative Haller index obtained from 27 patients who received a CT scan was 1.95 (range 1.54–2.45). All patients presented with cosmetic complaints and had not received any prior orthopedic treatment.

### Surgical technique

Preoperative measurement to determine the size of the orthopedic pectus bar was performed using the horizontal distance between the midaxillary line on both sides at the level of maximal protrusion of the sternum.

**Fig. 1:**
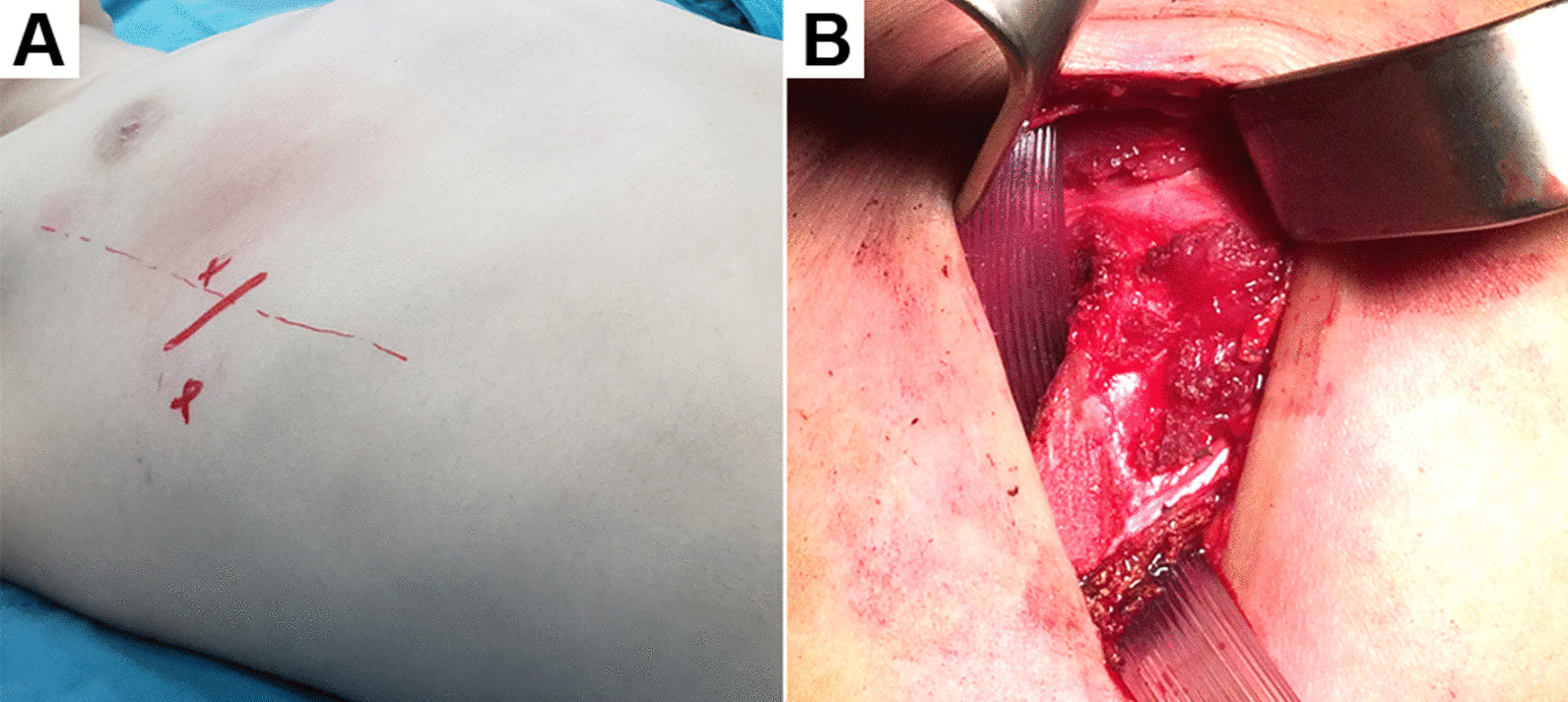
**a** Preoperative marking of the rib at the anterior axillary line. The upper and lower intercostal spaces were planned to divide. **b** A polyvinyl chloride tube was pulled out from the upper intercostal space to the lower intercostal space of the rib at the anterior axillary line

**Fig. 2:**
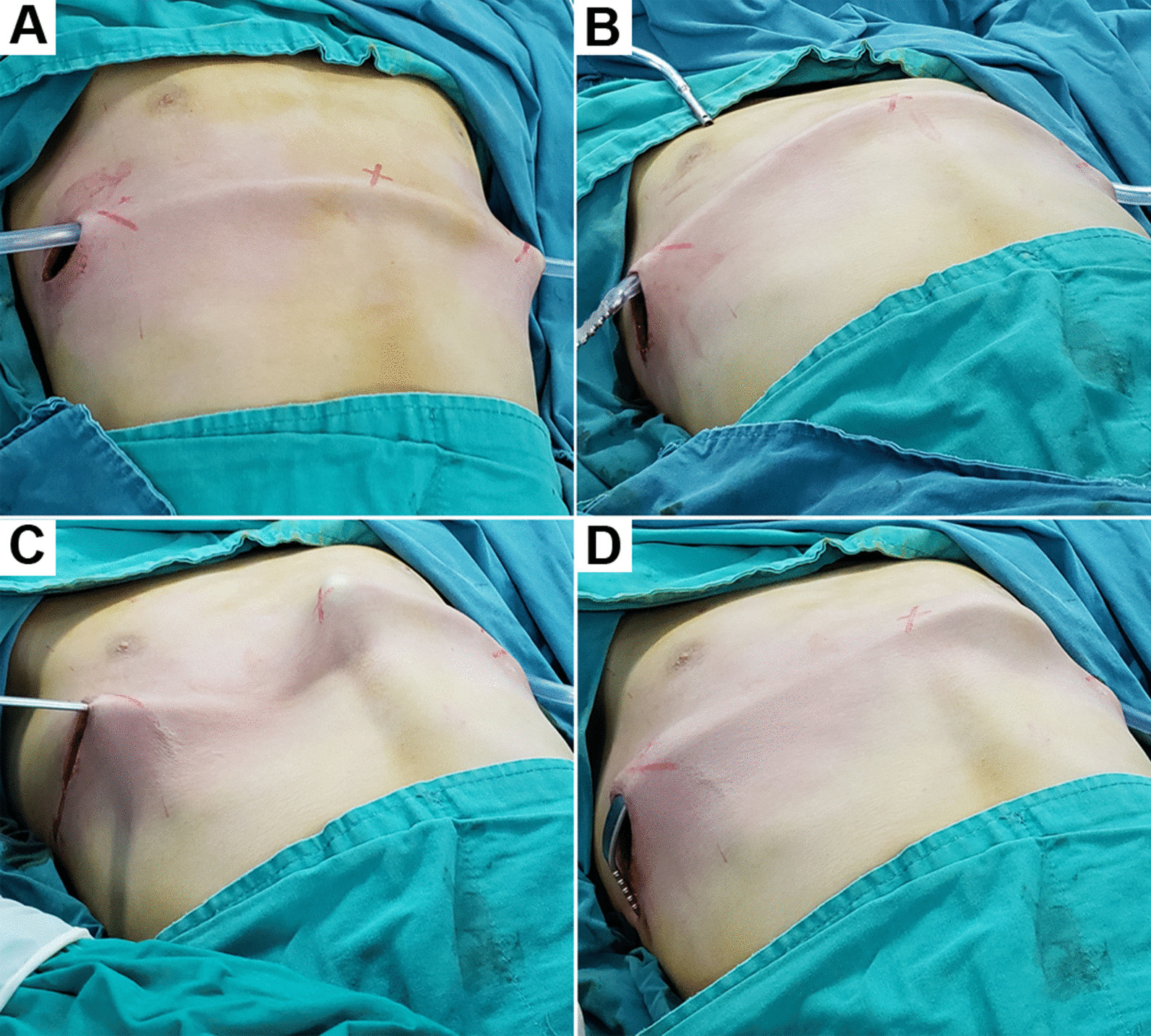
Operative procedures. **a** A PVC tube was passed through the subcutaneous tunnel from a skin incision to the contralateral incision. **b** One tip of the pectus bar was inserted into the PVC tube lumen. **c** The pectus bar was guided to pass through the subcutaneous by the PVC. **d** The pectus bar was flipped over 180° and advanced into and back out of the chest by passing from the upper intercostal space to the lower intercostal space. PVC: polyvinyl chloride

General anesthesia with endotracheal intubation was administered in all patients. The patient was positioned in the supine position with both arms abducted. Two transverse incisions, 3 cm each, were made on the midaxillary line at the level of the maximal protrusion (Fig. 1). Further dissection was performed to expose the rib at the anterior axillary line, of which the upper and lower intercostal spaces were each divided. Blunt dissection was performed subcutaneously towards the sternum via the incisions at both sides with a long-curved clamp, thus creating a subcutaneous tunnel. The pectus introducer passed through the subcutaneous tunnel from a skin incision to the contralateral incision (Fig. 2). A polyvinyl chloride (PVC) tube was attached to the introducer and guided through the subcutaneous tunnel, and then exited from both incisions. The 2 ends of the PVC tube were separately inserted into the upper intercostal space and pulled out from the lower intercostal space of the rib at the anterior axillary line. The pectus bar was modeled into a convex shape according to the particular characteristics of the deformity. One tip of the bar was inserted into the PVC tube lumen. With its convexity facing posteriorly, the bar passed under the selected rib through the intercostal space that had been prepared as described above. As the tip of the bar was being forwarded through the subcutaneous tunnel and reached the opposite side, the bar was flipped over 180° and advanced into and back out of the chest from the upper to the lower intercostal space of the rib at the anterior axillary line, with the guidance of the PVC tube. One or two stabilizers were fixed to the chest wall muscles with absorbable sutures. The lung was expanded under visual control before the closure of the thorax, and the wound was then closed in layers.

Posteroanterior and lateral chest X-rays were obtained 24 h after surgery to identify the bar location and to look for pneumothorax. Pain control was maintained with intravenous patient-controlled analgesia (IVPCA) using hydromorphone during the postoperative 48 h, followed by oral oxycodone. Antibiotics were used 48–72 h after surgery. Patients were advised to avoid contact sports within 3 months of surgery. Patient satisfaction was evaluated with the answers provided on questionnaires completed on postoperative day 3 and after bar removal.

### Statistical analysis

Normally distributed continuous data were expressed as mean ± standard deviation. Statistical analyses were performed using SPSS (ver. 20; SPSS, Inc., Chicago, IL, USA) for Windows.

## Results

**Fig. 3:**
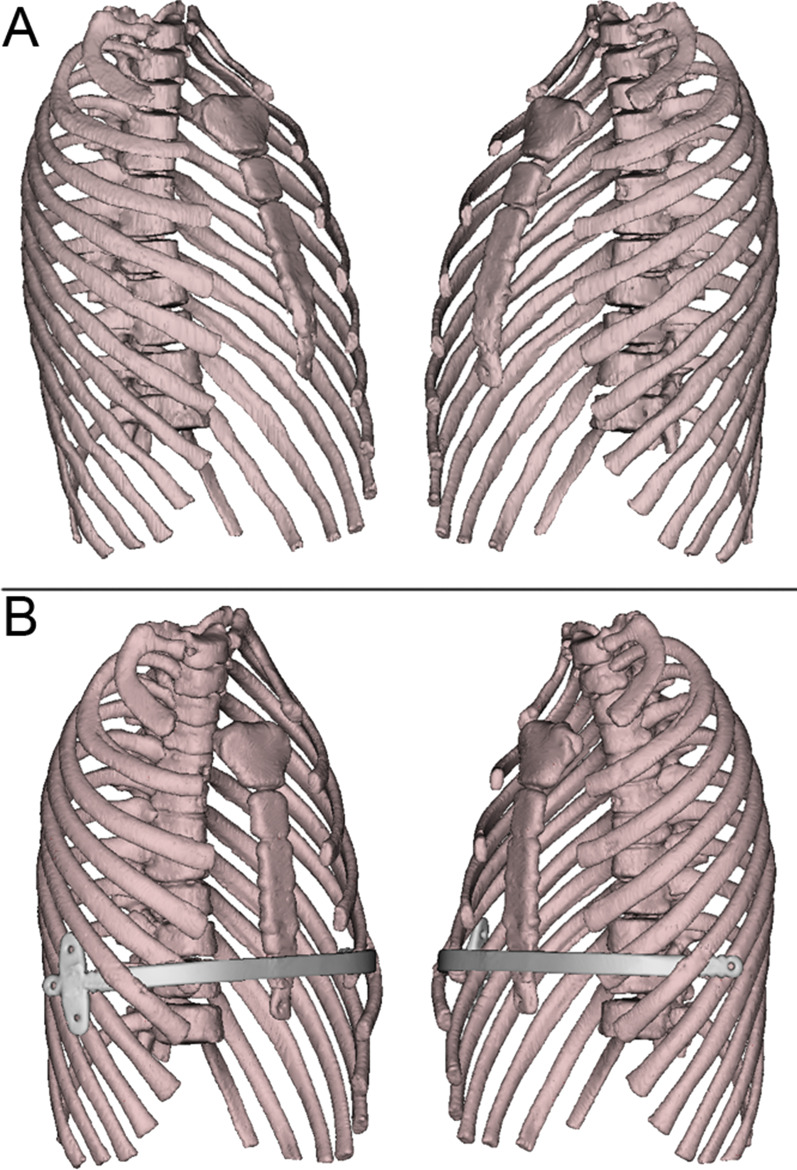
The 3-dimensional imaging of a 13-year-old patient’s computed tomography before **a** and after **b** the operation

**Fig. 4:**
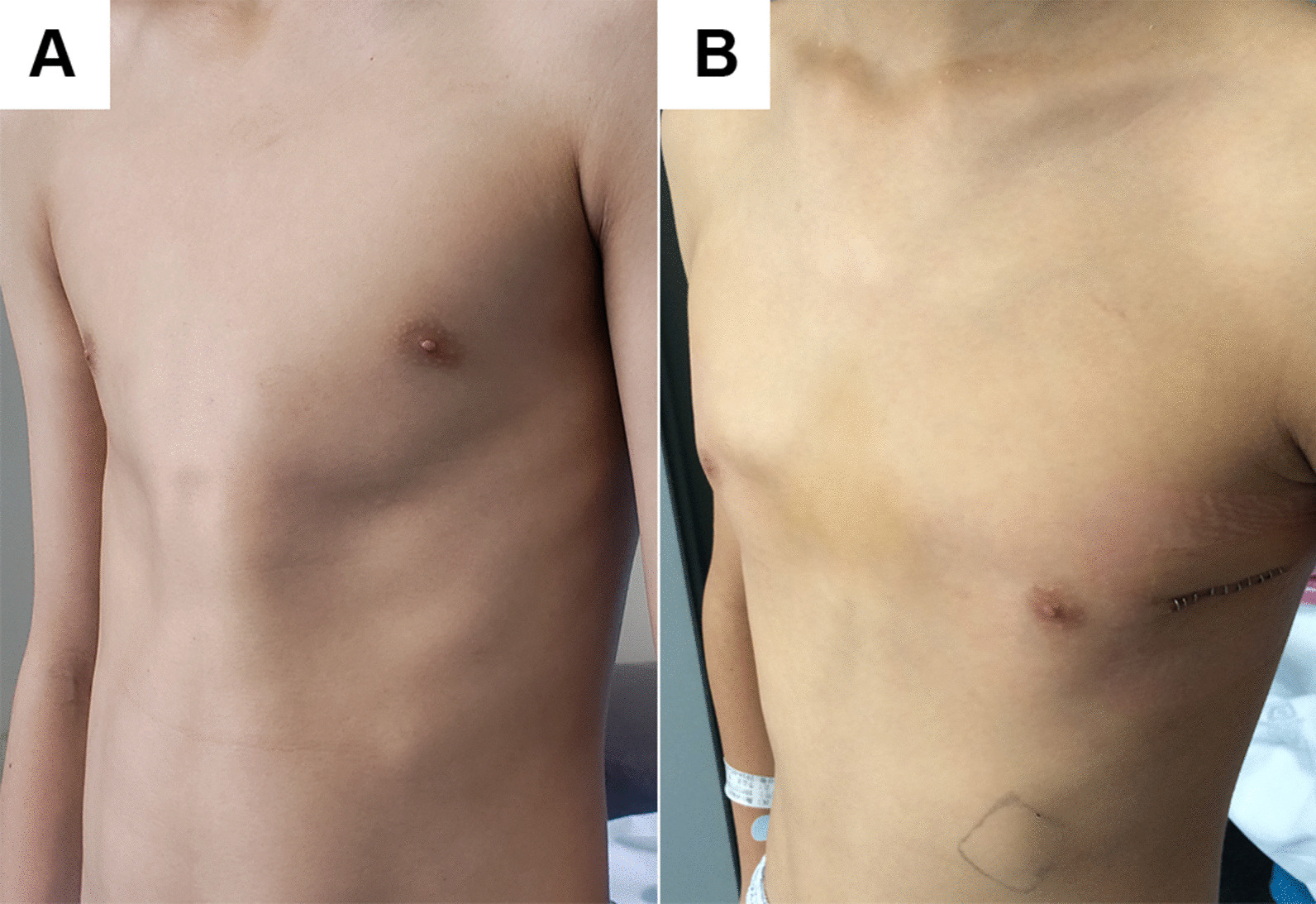
Symmetric pectus carinatum of a 13-year-old patient before **a** and after **b** surgery

All patients and/or their guardian reported excellent (n = 37) or good (n = 5) cosmetic results. A single bar was placed in every patient without any significant blood loss. In total, 2 stabilizers were equipped in 31 patients, while only 1 stabilizer was equipped in the other 11. The mean operation time and duration of postoperative stay were 87.14 min (range 55–125 min) and 4.05 days (range 1–12 days), respectively. The mean follow-up after the operation was 28.5 months (c 3–63 months). The 3-dimensional imaging and Symmetric pectus carinatum of a 13-year-old patient before and after the operation are shown in Figs. 3 and 4.

Wound infections occurred in 3 patients, with 2 being cured by antibiotics accordingly, and 1 presenting with persistent infection in the left incision, which resulted in the exposure of the implant. Despite antibiotic treatment and a debridement operation, the infection could not be controlled. The pectus bar in this patient was removed 4 months after the initial operation. Mild pneumothorax was diagnosed in 3 patients, but no drainage was required. One patient suffered from hydropneumothorax, which was treated with chest drainage.

There was no displacement of the bar during the follow-up period. A slight lateral shift of the bar occurred in 1 patient 6 weeks after the operation, but no recurrence of the malformation and no further shift were observed during the follow-up. All patients discontinued analgesics within 1 month after the operation without complaint of persistent pain. Finally, 20 patients underwent the removal of the bar between 2 and 3 years after surgery without recurrences of the malformation.

## Discussion

While the pathogenic mechanisms for PC remain unclear, the disease has a lower incidence rate than pectus excavatum and accounts for 6–22% of all chest wall deformities [2, 11]. It is more common in males with a male–female ratio of 4:1, and typically presents during childhood and aggravates dramatically during pubertal growth [12]. There are different types of PC according to the maximum point of the deformity: the chondrogladiolar form is the most common variety (involving the lower sternum) with symmetric or asymmetric protrusion, while the chondromanubrial form is much less common (involving the upper sternum) [13]. In contrast to pectus excavatum, pectus carinatum deformities are more evident and difficult to conceal with clothing or posture. As a result, the patients with PC suffer from poor self-confidence, feelings of inferiority, and are hesitant or refuse to participate in sports or social activities, which are all harmful to the patient's psychological health.

Treatment options for PC include nonoperative treatment and operative treatment. Nonoperative treatment for PC employs a specially designed orthotic chest brace to apply compression on the protruding sternum to achieve the correction of the deformity. This technique has been proven to be effective and associated with advantages of non-invasiveness and low morbidity [14, 15]. However, as it requires long-term application of the brace and does not immediately present a satisfactory result, non-compliance is frequent [16].

The conventional Ravitch operation and its modified procedures for PC [17, 18], consisting of open incision and resection of costal cartilages, are substantially invasive and associated with considerable postoperative pain and an obviously visible scar. The minimally invasive procedure for repair of PC, introduced by Abramson, has been performed widely with good cosmetic results and less postoperative complications. However, at least 4 ribs are involved in the fixation system, and postoperative wire breakage is difficult to avoid as the entire force working against the sternum is sustained by the wires. Although various modifications to the Abramson procedure have been applied, the essential principle of the surgery is still the same. Early wire breakage is the primary cause of bar displacement that results in orthopedic failure and re-operation. In addition, since both ends of the metal bars are fixed to the chest wall, the growth and development of the chest may be restricted.

In our technique, the pectus bar is placed over the sternum through the subcutaneous tunnel, with both ends passing through the intercostal spaces of the selected rib at the anterior axillary line. The protruding sternum is depressed by force being exerted by the selected ribs onto the bar. Since the intra- and extra-thorax placement of the bar results in an “autologous” force and stability, neither manual compression nor special wire fixation is needed. Furthermore, due to the exemption from the fixation of the bar to the bilateral ribs, this procedure can eliminate the restriction on the growth of the chest. Meanwhile, this procedure has an added advantage as the bar corrects the chest contour and simultaneously expands the chest laterally which produces a better cosmetic result. Finally, this operation can be performed with the conventional Nuss pectus bar and instrument, without any specially designed apparatus.

The most significant characteristic of our technique is that the bar passes through the thorax 4 times (2 intra- and 2 extra-thorax). The technique in principle is similar to the method proposed by Kálmán and Hock and their colleagues [19, 20], in which the bar is passed into or out of the chest parasternal using the finger for blind guidance. In contrast to this method, our procedure presented here is simpler and safer and does not entail a risk of injury to the heart or the great vessels, since the bar is passed through the thorax under direct vision. Compared with Tarhan’s technique [21], our procedure further simplifies the operation in the same safety setting and does not require a thoracoscope or presternal incision.

Unlike pectus excavatum, which is characterized by progressive aggravation before adolescence, the deformity of PC is not apparent in childhood but will be rapidly aggravated and reach its physically and psychologically disturbing peak during puberty. According to our experience, the best candidates for the minimally invasive correction are 12–18 years old. However, the decision of surgery is based on the flexibility of the chest wall rather than the age. Preoperative evaluation of the chest wall flexibility, by compression of the protruding area with the examiner’s hand, while the patient is taking a supine position or leaning against a wall, should be routinely performed for judging whether if a thorax is flexible enough to be corrected. The contraindications for a bar correction are non-malleable, rigid thoraces, as the chondromanubrial type of PC or very asymmetric deformities are more suitable for treatment with the open techniques [4, 19].

Eight patients with asymmetric deformity of chondrogladiolar type underwent a correction in our procedure and received good cosmetic results. Therefore, besides symmetric PC, we believe that this technique could also be considered a valid method for the correction of asymmetric PC in selected patients.

There was 1 case of uncontrolled wound infection, which ultimately resulted in the failure of the correction due to the removal of the pectus bar and stabilizers prior to the planned date. This should be ascribed to the insufficient coverage of the bar and stabilizer. The implants should be placed under and well covered by the muscle tissue to avoid subcutaneous exudate. Overall, we consider the morbidity of postoperative complications to be lower. The risk of wire breakage and bar displacement, as frequently observed in the Abramson technique, is avoided due to the fact that the implant is not fixed to the ribs.

Taken together, compared with Abramson procedure and other techniques, the advantages of our procedure are that there are (i) no complications with wire breakage or bar displacement, (ii) no potential restriction on the growth of the chest wall, (iii) no risk of injury to the heart or great vessels; and that (iv) the chest wall can be expanded laterally for a better appearance.

This study has several limitations. First, this is a retrospective study from a single center, which is limited by the small sample size. Surgical skill level needs to be considered in a study of this nature. However, this new minimally invasive technique for 42 patients with PC in our hospital was performed by 3 different skilled surgeons with excellent technique. Given that satisfactory cosmetic results were obtained in all the patients, we believe it is worth spreading this safe and feasible technique for chondrogladiolar PC in other centers. Second, all the patients enrolled in the study were chondrogladiolar PC and only 20 patients underwent the removal of the bar, further experience with more patients is necessary to evaluate the long-term results and to study whether this technique is applicable to other forms of PC.

## Conclusion

In conclusion, this preliminary study demonstrated that the new technique for minimally invasive correction of PC deformity is a safe and feasible procedure yielding good results and minimal complications.

## Data Availability

All data generated or analyzed during this study are included in this article.

## Abbreviations

PC: Pectus carinatum
ECG: Electrocardiography
CT: Computed tomography
PVC: Polyvinyl chloride
IVPCA: Intravenous patient-controlled analgesia
